# Irritable Bowel Syndrome and Me: A Sworn Rivalry

**DOI:** 10.31729/jnma.6067

**Published:** 2021-03-31

**Authors:** Selika Shakya

**Affiliations:** 1 Kathmandu Medical College and Teaching Hospital, Sinamangal, Kathmandu, Nepal

**Keywords:** *irritable bowel syndrome*, *medical student*, *Nepal*

## Abstract

“Three more hours”, I tell myself as I find my way to the exam hall. I see a bunch of my classmates cramming for the last minute, and here I am, in a corner, reminding myself that it's just three more hours till I get away from this horror. Irritable Bowel Syndrome is a gastrointestinal disorder in which there is dull achy lower abdominal pain associated with either diarrhea or constipation. Though it has high prevalence, it is still an underrated disease which can be easily missed in clinical settings.

“Three more hours”, I tell myself as I find my way to the exam hall. I see a bunch of my classmates cramming for the last minute, and here I am, in a corner, reminding myself that it's just three more hours till I get away from this horror. As my brain tries to recall what I've studied for the exam, and calm myself down, my abdomen says otherwise and lets out a groaning pain, making me panic all over again. With a bloated abdomen and loads of discomfort, halfheartedly, I take my seat. Yes, this is me during my most stressful hours, moments before my exams.

I've had abdominal pain during my exams for as long as I could remember. The first episode was when I was 9 years old. Maybe that was when I realized that examinations were stressful. It started as a dull pain which hyped during the first and second hours of the three-hour exam and gradually diminished as the exam came to an end. As a 9-year-old kid, I couldn't really explain what had just happened to me. I couldn't tell my parents because it didn't hurt anymore, not until the next exam, when the same thing happened to me.

The first time I ever told my parents was when the pain was really unbearable and I couldn't concentrate on my exam. And that's probably why my performance started degrading. Even though I studied really hard for the exams, I just couldn't do well, because of the pain. And the worst thing was that the pain disappeared when the exam was over so I couldn't tell the doctors where it'd hurt. My parents thought that I was making things up because I couldn't do well in the exams. I guess assuming ten-year-old kids lying about having stomach ache was easier than believing my story of vanishing pain but I wasn't lying. After that, no matter how much it hurt, I never told anyone about it.

It's been almost twelve years since that happened. And guess what, nothing has changed. I still have abdominal pain during my worst hours. As a student, I'm most stressed during the exams and Irritable Bowel Syndrome comes as an uninvited guest, leaving me with pain and misery.

During my second year at Kathmandu Medical College, I was excited to learn about the Gastrointestinal System. I thought, maybe I could get some answers, for I didn't know what disease I had. I just knew that I had bad stomach aches that stormed during the exam period and waned away after the exams ended, as an invisible enemy. So, what exactly did I have, I didn't know. I read almost all the chapters there were, in my pathology book. None of my symptoms made sense until I finally came across one disease that fit all the puzzles into place. Irritable Bowel Syndrome (IBS), at last, I found you!

So, what is IBS? Irritable Bowel Syndrome is a functional bowel disorder characterized by chronic abdominal pain or discomfort with alternation in bowel habits in the absence of any pathological condition.^1^ To be diagnosed as IBS, there are certain criteria that are got to be fulfilled called Rome III Criteria. The Rome III Criteria states that; the patient should have recurrent abdominal pain or discomfort for at least 3 days/month in the last 3 months, associated with two or more of the following features: improvement with defecation, onset associated with a change in the frequency of stool or onset associated with a change in the form of stool.^2^ In my case, all of the criteria checked out.

As appalled as I was when I found out about this problem of mine, I was devastated to also discover that IBS had no permanent cure. The temporary treatment focused on relieving the symptoms by managing diet and lifestyle. Some studies have shown that diet modification by strictly undertaking Fermentable Oligosaccharides, Disaccharides, Monosaccharides, and Polyols (FODMAP) diet,^3^ regular exercise and relaxation therapies help relieve some of the IBS symptoms. For me, I had no luck with any of those. In addition to the non-pharmacological therapies, certain medications like fiber supplements, Lubiprostone for constipation,^4^ anti-diarrheal for diarrhea dominant IBS, tricyclic antidepressants (TCAs), and selective serotonin receptor inhibitors (SSRIs) have been prescribed to IBS patients.^5^ But the thing about the IBS population is that it is diverse with each individual, presenting with different complaints, hence no drug can be a perfect solution for all.

The prevalence of IBS globally is 11.4% with female predominance and yet many physicians fail to recognize it. There are many who are either undiagnosed or even misdiagnosed. IBS can be easily missed beneath the pile of other gastrointestinal diseases. This may be an underrated disease but it's dominating my life showering me with unfortunate circumstances. But then again, I've learned to live with it. The new advanced clinical trials and modern therapies to combat this disease do look promising, but will they finally put down the horror of IBS is what is yet to be figured out. But until then, IBS is gonna keep wreaking havoc into my life....... continuing this sworn rivalry.
